# The Medical Bill

**Published:** 1894-12

**Authors:** 


### THE MEDICAL BILL.

At this writing the medical bill awaits consideration in the Senate. It came up on its final reading in the House November 21st, and passed with the aid of the Speaker's vote. The vote stood eighty-seven for, and sixty-five against, the bill. Eighty-eight is the necessary legal majority in the House. This necessary vote was cast by Speaker Fleming, who has been a warm friend of the bill from the first. Major Fouché, of Rome, and Mr. Branch made good speeches for the bill. The opponents secured a reconsideration for the purpose of amendment, and the reconsideration was appointed for November 24th. The most objectionable part of the bill seemed to be that clause relative to the meeting of the board on the second Tuesday in April. So an amendment was inserted at this place providing that this meeting shall be held at such a time near the close of the session as may be agreed upon by the boards of examiners and the faculties of the colleges.

On the bill, thus amended, the vote was ninety-two to none (unanimous vote). There is every reason to believe that the bill will pass the Senate with very little opposition.

